# Left ventricular T1-mapping in diastole versus systole in patients with mitral regurgitation

**DOI:** 10.1038/s41598-022-23314-6

**Published:** 2022-11-21

**Authors:** Boyang Liu, Harish Sharma, Kyaw Su Khin, Roman Wesolowski, Sandeep S. Hothi, Saul G. Myerson, Richard P. Steeds

**Affiliations:** 1 Institute of Cardiovascular Sciences, University of Birmingham, Edgbaston, Birmingham, B15 2TT UK; 2 Department of Cardiology, Queen Elizabeth Hospital, University Hospitals Birmingham NHS Foundation Trust, Birmingham, B15 2TH UK; 3 Institute of Translational Medicine, University Hospitals Birmingham, Birmingham, UK; 4 Royal Wolverhampton NHS Hospitals Trust, Wolverhampton Road, Wolverhampton, WV10 0QP UK; 5 Division of Cardiovascular Medicine, Radcliffe Department of Medicine, University of Oxford, Oxford, UK

**Keywords:** Magnetic resonance imaging, Translational research

## Abstract

Cardiovascular magnetic resonance T1-mapping enables myocardial tissue characterisation, and is capable of quantifying both intracellular and extracellular volume. T1-mapping is conventionally performed in diastole, however, we hypothesised that systolic readout would reduce variability due to a reduction in myocardial blood volume. This study investigated whether T1-mapping in systole alters T1 values compared to diastole and whether reproducibility alters in atrial fibrillation compared to sinus rhythm. We prospectively identified 103 consecutive patients recruited to the Mitral FINDER study who had T1 mapping in systole and diastole. These patients had moderate or severe mitral regurgitation and a high incidence of ventricular dilatation and atrial fibrillation. T1, ECV and goodness-of-fit (R^2^) values of the T1 times were calculated offline using Circle cvi42 and in house-developed software. Systolic T1 mapping was associated with fewer myocardial segments being affected by artefact compared to diastolic T1 mapping [217/2472 (9%) vs 515/2472 (21%)]. Mean native T1 values were not significantly different when measured in systole and diastole (985 ± 26 ms vs 988 ± 29 respectively; *p* = 0.061) and mean post-contrast values showed similar good agreement (462 ± 32 ms vs 459 ± 33 respectively, *p* = 0.052). No clinically significant differences in ECV, native T1 and post-contrast T1 were identified between diastolic and systolic T1 maps in males versus females, or in patients with permanent atrial fibrillation versus sinus rhythm. A statistically significant improvement in R^2^ value was observed with systolic over diastolic T1 mapping in all analysed maps (n = 411) (96.2 ± 1.4% vs 96.0 ± 1.4%; *p* < 0.001) and in subgroup analyses [Sinus rhythm: 96.1 ± 1.4 vs 96.3 ± 1.4 (n = 327); *p* < 0.001. AF: 95.5 ± 1.3 vs 95.9 ± 1.2 (n = 80); *p* < 0.001] [Males: 95.8 ± 1.4 vs 96.1 ± 1.3 (n = 264); *p* < 0.001; Females: 96.2 ± 1.3 vs 96.4 ± 1.4 (n = 143); *p* = 0.009]. In conclusion, myocardial T1 mapping is associated with similar T1 and ECV values in systole and diastole. Furthermore, systolic acquisition is less prone to gating artefact in arrhythmia.

## Introduction

Cardiovascular magnetic resonance (CMR) is the primary non-invasive imaging modality for myocardial tissue characterisation, utilising a number of different imaging techniques. Myocardial T1 mapping is capable of quantifying both intracellular and extracellular myocardial changes based on T1 and extracellular volume (ECV) measurements^1^ and is increasingly used for diagnosis of disease. Parametric T1 mapping values correlate favourably with histological fibrosis observed on myocardial biopsy^2^ and are markers of adverse myocardial remodelling. Parametric T1 mapping has incremental value in predicting outcomes, including arrhythmia and mortality. The technique has proven clinical utility in cardiac amyloidosis, iron deposition, Fabry disease, myocarditis, as well as potential clinical utility in the assessment of ischaemia and non-ischaemic cardiomyopathy, heart failure, congenital heart disease and suspected transplant rejection^1^.

T1 mapping images are traditionally acquired in end-diastole. T1 relaxation times and ECV values are then calculated from the signal of the myocardium, carefully selected to exclude voxels at the endocardial order, thus minimising contamination from the blood due to partial volume effect. However, although myocardial motion is lowest in end-diastole (hence the usual choice of this point in the cardiac cycle)^3^, myocardial thickness is also lowest at this time. Furthermore, the high prevalence of ectopy and arrhythmia (e.g. AF) in patients with cardiac disease, cause electrocardiogram (ECG) gating artefacts^4^ that adversely affect acquisition sequences such as the 3(3)3(3)5 scheme MOLLI, which requires a consistent R-R interval over its 11 cardiac cycle acquisition period^5^. T1 mapping in systole is feasible^6^ and may help to overcome these issues. This could potentially negate gender-related differences in ECV^7–9^ and myocardial blood volume, which is lowest in peak systole^10^.

We therefore hypothesised that compared to diastolic T1 mapping, systolic T1 mapping will: (i) provide more reliable ECV values by measuring at a time when intramyocardial vascular space and capillary blood pool are smallest; (ii) reduce gating artefacts as reflected by higher R^2^ values; (iii) reduce the variation in ECV and T1 results related to patient gender and presence of AF.

We chose to examine this hypothesis in patients with moderate and severe mitral regurgitation (MR). This population has a high prevalence of atrial fibrillation (AF) and ventricular dilatation.

## Methods

### Study population

The present study is a planned sub-study of patients recruited to the Mitral FINDER project, for which the full study protocol has been previously published^11^. In brief, mitral FINDER was a multicentre prospective study of adult patients (aged 18 and over) with primary degenerative MR, recruited from 3 regional tertiary cardiac centres. Mitral regurgitation was diagnosed and quantified by a multiparametric echocardiographic approach, as recommended by international guidelines^12,13^. For the present study, the pre-operative CMR studies of patients recruited for the mitral FINDER project at the Queen Elizabeth Hospital Birmingham were retrospectively assessed. Patients were included if native and post-contrast T1 values were acquired in diastole and systole.

Exclusion criteria included patients without T1 values acquired in both diastole and systole, non-degenerative MR aetiology, co-existing moderate or severe aortic valve disease, congenital disease, inherited or acquired cardiomyopathy, symptomatic coronary artery disease, uncontrolled AF (resting heart rate > 100/min), pregnancy, and those unable to undergo CMR. All included AF patients were in AF at the time of the scan (permanent AF).

### T1 mapping

All patients underwent CMR at 1.5 T at University Hospital Birmingham (1.5 Tesla scanner Magnetom Avanto, Siemens) using an 18-channel phased-array coil with the participant supine. For myocardial characterisation, myocardial and blood pool relaxation times were measured using the modified Look-Locker inversion recovery (MOLLI) sequence^14^ pre- and 15 min after the administration of gadolinium contrast, at the basal and mid left ventricular short axis level (3(3)3(3)5 scheme). Echo time (TE) was 1.01 ms for 4-chamber (4Ch) and 1.06 ms for short axis slices in both systole and diastole. The echo repetition time (TR) was 338 ms in systole and varied in diastole depending on the heart rate (normally above 750 ms). Flip angle was 35° and voxel size was 2 × 2 × 8 mm^3^ and 1.8 × 1.8 × 8 mm^3^ for 4ch and short axis slices respectively. The first inversion time was 100 ms for all acquisitions, partial acceleration factor 2 (36 reference lines) and partial Fourier imaging factor 7/8. In systole, the minimum trigger delay was set to 0 ms, with a data acquisition at 257.5 ms. In diastole, the trigger delay was variable depending on the heart rate (normally above 650 ms). There were 92 readouts per heartbeat. MOLLI T1-maps were acquired from vendor-provided product protocols (MyoMaps, Siemens Heallthcare, Erlangen, Germany) which provides pixel-based myocardial quantification and colour mapping with motion correction. T1 times and ECV were calculated offline using Circle cvi42^®^ (Circle Cardiovascular Imaging Inc. Canada), in accordance with the European Society of Cardiology recommendations^15^. A 20% offset was used on endocardial and epicardial contours to avoid the blood-myocardial boundary. Extracellular volume was calculated using blood and myocardial T1 values pre- and post-contrast using the validated formula below:

$${\text{ECV}} = \left( {\frac{{\frac{1}{{{\text{T}}1_{{{\text{myo}}\;{\text{post}}}} }} - \frac{1}{{{\text{T}}1_{{{\text{myo}}\;{\text{pre}}}} }}}}{{\frac{1}{{{\text{T}}1_{{{\text{blood}}\;{\text{post}}}} }} - \frac{1}{{{\text{T}}1_{{{\text{blood}}\;{\text{pre}}}} }}}}} \right) \times (1 - {\text{haematocrit}})$$

Haematocrit was measured at the same time as the CMR study. DICOM images were imported into in-house software (MIPPY 2, version 21.2.1) to create goodness-of-fit (R^2^) values based on the difference between the measured and fitted inversion recovery signal curves of the 11 consecutive images acquired by the 3(3)3(3)5 scheme in the basal and mid-left ventricular level, pre- and post-contrast. Inversion recovery signal curves were generated based on the exponential model. Baseline LV parameters such as volume, mass and function were obtained using CMR.

### Statistical analysis

Normal and non-normally distributed continuous variables are expressed as mean ± standard deviation or median with interquartile range and were compared using Student’s T test or Mann–Whitney U test respectively. Linear regression (R^2^) values of native and post-contrast T1 times at the basal and mid-left ventricular levels (n = 411) were analysed using paired Student’s T test. Categorical variables were compared using a chi squared test. A *p* value of < 0.05 was considered significant. All analyses were conducted using SPSS software version 23.0 (SPSS, Inc, Chicago, IL, USA).

### Ethical approval and consent to participate

The study was approved by the ethical committee of the UK National Research Ethics Service (15/EM/0243) and conformed to the Helsinki Declaration. Subjects gave written informed consent to participate.

## Results

### Baseline characteristics

Amongst 150 patients recruited to the mitral FINDER study, 103 patients underwent T1 mapping in systole and diastole and were included in this study. Baseline demographics are shown in Table 1. Patients had a mean age of 62 ± 15 years and were predominantly male [66/103 (64%)] with a mean body mass index of 25 ± 4 kg/m^2^. Prior diagnoses included AF 20/103 (19%), diabetes 2/103 (2%) and hypertension 34/103 (33%). Patients had a mean New York Heart Association (NYHA) classification of 1.4 ± 0.6, median estimated glomerular filtration rate (eGFR) of 74 ml/min/1.73m^2^ (IQR: 63–86) and median NT-proBNP level of 203 ng/L (IQR: 108–596).

**Table 1:** Baseline demographics of the population studied, including subgroups (males, females, AF and sinus rhythm).

	All patients (N = 103)	Males (N = 66)	Females (N = 37)	*P* value	Permanent AF (N = 20)	Sinus Rhythm (N = 83)	*P* value (95% CI)
Mean age (years)	62 ± 15	64 ± 13	58 ± 17	0.055	70 ± 9	60 ± 16	0.008*
Male Sex	66 (64%)	–	–	–	14 (70%)	53 (64%)	0.605
BMI (kg/m^2^)	25 ± 4	26 ± 4	24 ± 5	0.029*	27 ± 4	25 ± 4	0.047*
Smoking history	41 (40%)	31 (46%)	9 (28%)	0.068	8 (40%)	33 (40%)	0.984
Permanent AF	20 (19%)	14 (21%)	6 (17%)	0.605	20 (100%)	0 (0%)	–
Diabetes	2 (2%)	0 (0%)	2 (6%)	–	0 (0%)	2 (2%)	–
Hypertension	34 (33%)	25 (33%)	9 (25%)	0.205	9 (45%)	25 (30%)	0.204
NYHA class	1.4 ± 0.6	1.4 ± 0.6	1.5 ± 0.7	0.449	1.7 ± 0.7	1.4 ± 0.6	0.055
eGFR (ml/min/1.73m^2^)	74 (63–86)	76 (65–84)	71 (57–91)	0.637	58 (51–75)	78 (67–87)	0002*
NT-proBNP (ng/L)	203 (108–596)	211 (93–474)	195 (108–812)	0.568	2161 (837–2463)	174 (70–279)	< 0.001*
LVEDVi (ml/m^2^)	99 ± 24	106 ± 24	87 ± 20	< 0.001*	102 ± 30	98 ± 23	0.451
LVESVi (ml/m^2^)	32 ± 12	34 ± 12	29 ± 10	0.026*	41 ± 14	30 ± 10	< 0.001
LVEF (%)	68 ± 8	68 ± 8	67 ± 7	1.00	60 ± 14	70 ± 7	< 0.001*
LVMi (g/m^2^)	65 ± 14	71 ± 12	54 ± 12	< 0.001*	67 ± 14	65 ± 15	0.508
Haematocrit (L/L)	0.40 ± 0.04	0.40 ± 0.04	0.39 ± 0.04	0.227	0.40 ± 0.04	0.40 ± 0.04	1.0

Values represent mean ± standard deviation or median (IQR).

*BMI* body mass index, *AF* atrial fibrillation, *NYHA* New York Heart Association, *eGFR* estimated glomerular filtration rate, *NT-proBNP* N-terminal pro B-type natriuretic peptide.

*P* values with a * denotes statistical significance.

### Systolic versus diastolic T1 mapping in the overall cohort

**Figure 1:**
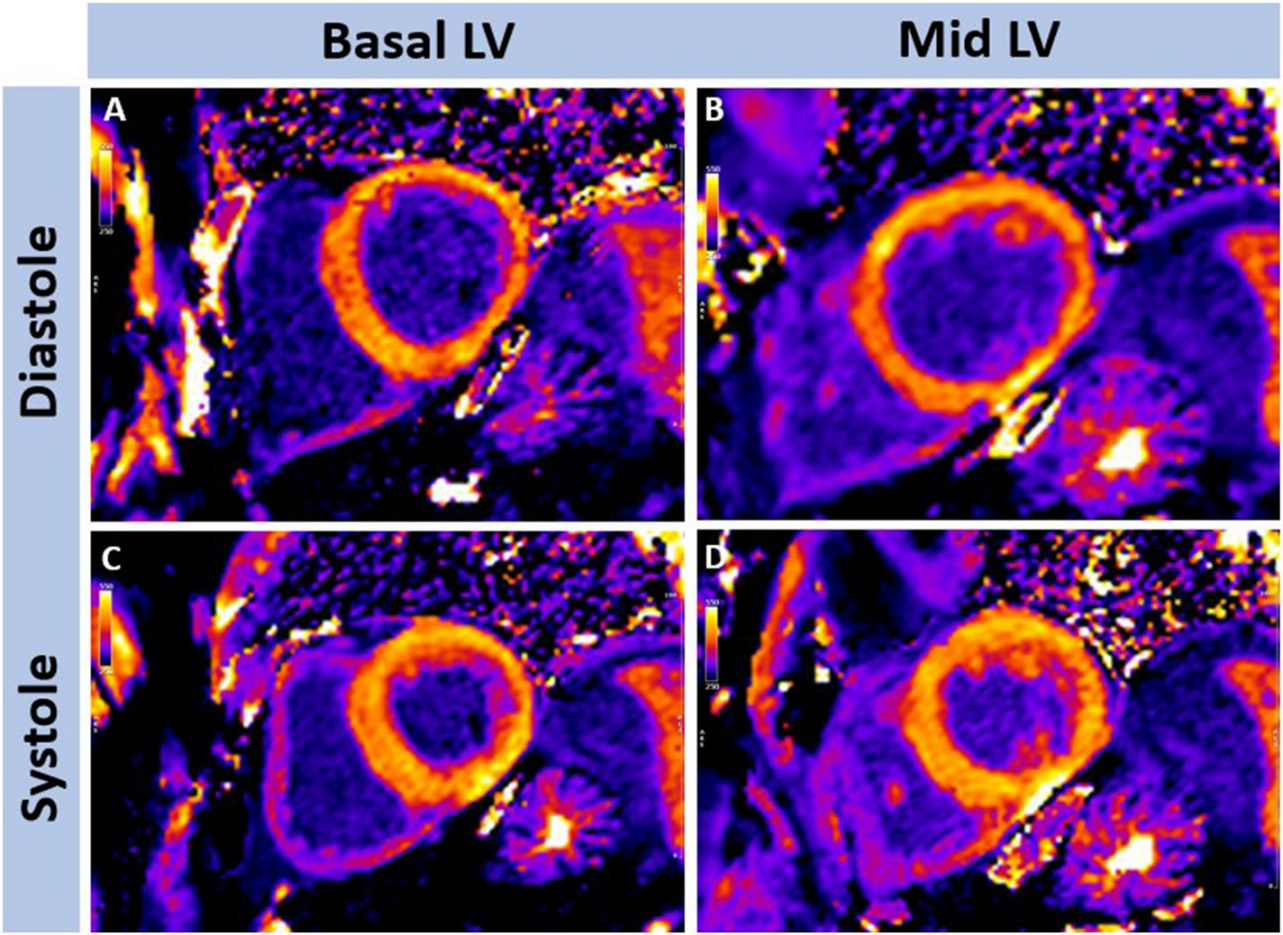
Examples of differences in left ventricular (LV) myocardial thickness following T1 mapping within the same patient in diastole and systole at the basal (**A** + **C**) and mid (**B** + **D**) left ventricular level in diastole (**A** + **B**) and systole (**C** + **D**), post-contrast.

**Figure 2:**
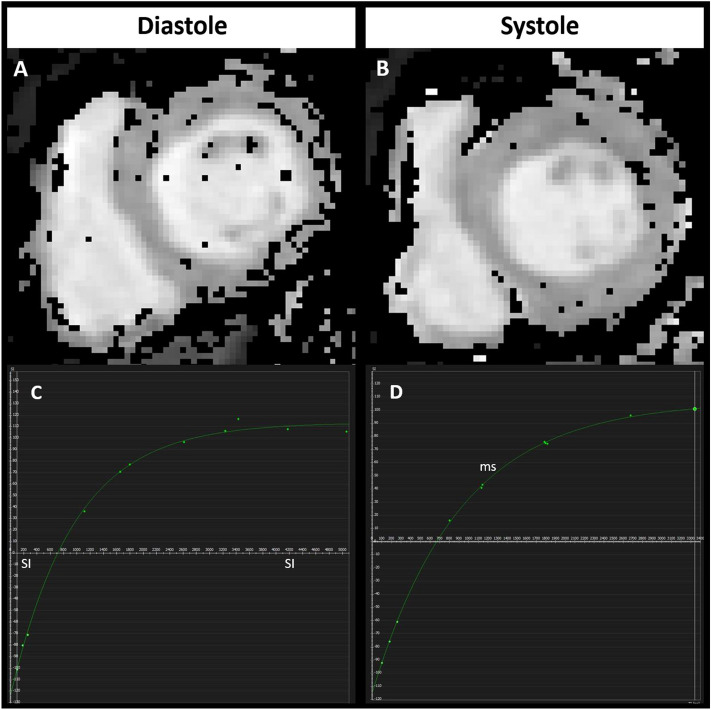
Examples showing how goodness-of-fit R^2^ values were generated in diastole (**A** + **C**) and systole (**B** + **D**) by in-house software. Graphs (**C** + **D**) demonstrate the greater difference in measured T1 values to the fitted curve in diastole (**C**) compared to systole (**D**).

**Figure 3:**
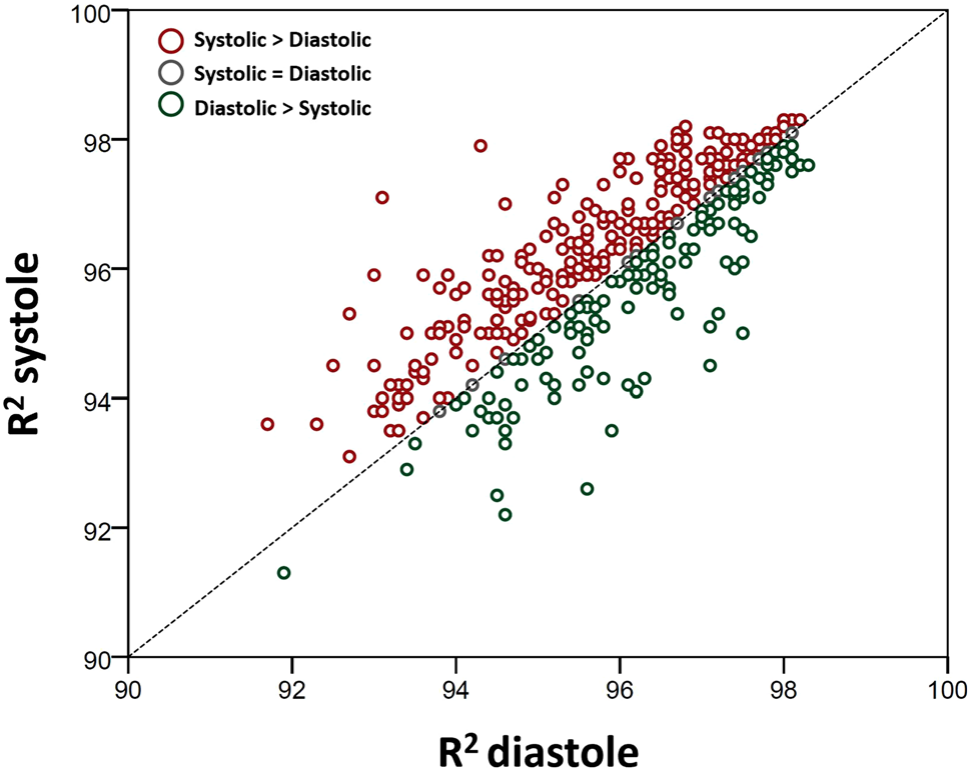
A graph of R^2^ values plotted in diastole and systole, showing excellent correlation (r = 0.8) and low variability between the methods.

Examples of the difference between diastolic and systolic myocardial thickness are demonstrated in Fig. 1. All T1 maps were checked for gating, wrap, breathing, motion correction and susceptibility artefacts prior to inclusion. In total, 9% (217/2472) of systolic segments and 21% (515/2472) of diastolic T1 map segments were excluded. Analysis of the difference between the measured signal curve and the fitted curve (R^2^), showed a significantly greater mean R^2^ value (96.2 ± 1.4 vs 96.0 ± 1.4; *p* < 0.001) with systolic imaging (an example is shown in Fig. 2). Further analysis found that systolic R^2^ was higher in 62% of cases, whilst diastolic measurement was higher in 32% (6% identical), shown in Fig. 3. However, the difference between systolic and diastolic T1 mapping was not clinically significant, as systolic T1 maps had equivalent mean native values compared to diastolic measurement (985 ± 26 ms vs 988 ± 29 respectively, *p* = 0.061) and the same was true for mean post-contrast values (462 ± 32 ms vs 459 ± 33 respectively, *p* = 0.052)—Table 2. Although there was a trend towards a very small difference (3 ms), this was not considered to be clinically meaningful. Similar results were observed when repeating the analysis replacing global T1 values with mid-septal T1 values obtained from a region of interest approach (Supplementary Table 1).

**Table 2:** T1 mapping—differences between diastole and systole.

		Diastole		95% Confidence interval	Lower	Upper	*P* Value
		N	Mean ± Std deviation	Mean ± Std deviation			
All	Native T1 (ms)	202	988 ± 29	985 ± 26	− 0.14	6.14	0.061
	Post-contrast T1 (ms)	199	459 ± 33	462 ± 32	− 4.55	0.02	0.052
	R^2^	411	96.0 ± 1.4	96.2 ± 1.4	− 0.34	− 0.18	< 0.001*
Males	ECV fraction (%)	129	26.5 ± 2.9	26.6 ± 2.5	− 0.42	0.20	0.489
	iECV (%/m^2^)	129	18.9 ± 3.8	19.0 ± 3.8	− 0.30	0.14	0.482
	Native T1 (ms)	131	984 ± 27	982 ± 26	− 1.61	6.15	0.251
	Post-contrast T1 (ms)	128	470 ± 25	470 ± 22	− 2.64	1.63	0.640
	R^2^	264	95.8 ± 1.4	96.1 ± 1.3	− 0.39	− 0.19	< 0.001*
Females	ECV fraction (%)	70	28.5 ± 3.0	28.4 ± 2.7	− 0.37	0.62	0.625
	iECV (%/m^2^)	70	15.3 ± 3.8	15.3 ± 3.8	− 0.25	0.30	0.852
	Native T1 (ms)	71	996 ± 32	991 ± 25	− 1.07	9.77	0.115
	Post-contrast T1 (ms)	71	440 ± 38	446 ± 40	− 10.55	− 0.33	0.037*
	R^2^	143	96.2 ± 1.3	96.4 ± 1.4	− 0.05	− 2.65	0.009*

A comparison of mean T1 times and R^2^ values in systole and diastole of all patients and of males and females to examine difference in sexes.

*P* values with a * denotes statistical significance.

Intra and inter-observer variability when measuring T1 values in systole and diastole was tested in a random sample of 10 patients but differences were small and were not statistically significant (Supplementary Table 2, Supplementary Figs. 1 and 2).

### T1 mapping in diastole and systole: differences between sexes

Compared to males, females had higher ECV in both diastole [28.5 ± 2.8 vs 26.6 ± 3.0%; *p* < 0.001] and systole [28.2 ± 2.8 vs 26.7 ± 2.5%; *p* < 0.001]. Similarly, females possessed higher native T1 times, and lower post-contrast T1 relaxation time. Compared to diastolic T1 mapping, the use of systolic imaging did not significantly affect ECV values [Males: 26.5 ± 2.9 vs 26.6 ± 2.5%; *p* = 0.489. Females: 28.5 ± 3.0 vs 28.4 ± 2.7%; *p* = 0.625] or alter associated native T1 times [Males: 984 ± 27 vs 982 ± 26 ms; *p* = 0.251. Females: 996 ± 32 vs 991 ± 25 ms; *p* = 0.115]. Sex-related differences between diastolic and systolic mapping are shown in Table 2. Systolic T1 mapping produced marginally higher post-contrast T1 relaxation times in females [440 ± 38 vs 446 ± 40 ms; *p* = 0.037] but not in males [470 ± 25 vs 470 ± 22 ms; *p* = 0.640]. In both sexes, there was a statistically significant improvement in R^2^ value with systolic T1 mapping.

### T1 mapping in diastole and systole: differences between sinus rhythm and AF

Aggregated basal and mid-left ventricular T1 maps from patients with atrial fibrillation (n = 50) had slightly higher ECV values than patients in sinus rhythm (n = 207), when imaged in both diastole (28.7 ± 2.7 vs 27.0 ± 3.1%, *p* < 0.001) and systole (28.7 ± 2.6 vs 26.9 ± 2.6%, *p* < 0.001). No significant differences in absolute ECV, native T1 and post-contrast T1 values were identified between diastolic versus systolic T1 maps in patients with permanent atrial fibrillation or sinus rhythm (Table 3). However, for both groups, there was a statistically significant improvement in R^2^ value with systolic T1 mapping.

**Table 3:** T1 mapping in AF and sinus rhythm: differences between diastole and systole.

		N	Diastole	Systole	95% Confidence interval		*P* value
			Mean ± Std deviation	Mean ± Std deviation	Lower	Upper	
SR	ECV fraction (%)	160	26.9 ± 3.1	26.9 ± 2.6	− 0.29	0.31	0.961
	Native T1 (ms)	163	985 ± 28	983 ± 24	− 0.53	5.99	0.100
	Post-contrast (ms)	160	461 ± 31	462 ± 31	− 3.25	1.10	0.331
	R^2^	327	96.1 ± 1.4	96.3 ± 1.4	− 0.30	− 0.12	< 0.001*
AF	ECV fraction (%)	39	28.6 ± 2.5	28.8 ± 2.6	− 0.73	0.39	0.548
	Native T1 (ms)	39	1000 ± 30	996 ± 31	− 5.11	13.36	0.372
	Post-contrast (ms)	39	452 ± 43	459 ± 38	− 14.70	0.40	0.063
	R^2^	80	95.5 ± 1.3	95.9 ± 1.2	− 0.65	− 0.25	< 0.001*

*ECV* extracellular volume.

*P* values with a * denotes statistical significance.

## Discussion

**Figure 4:**
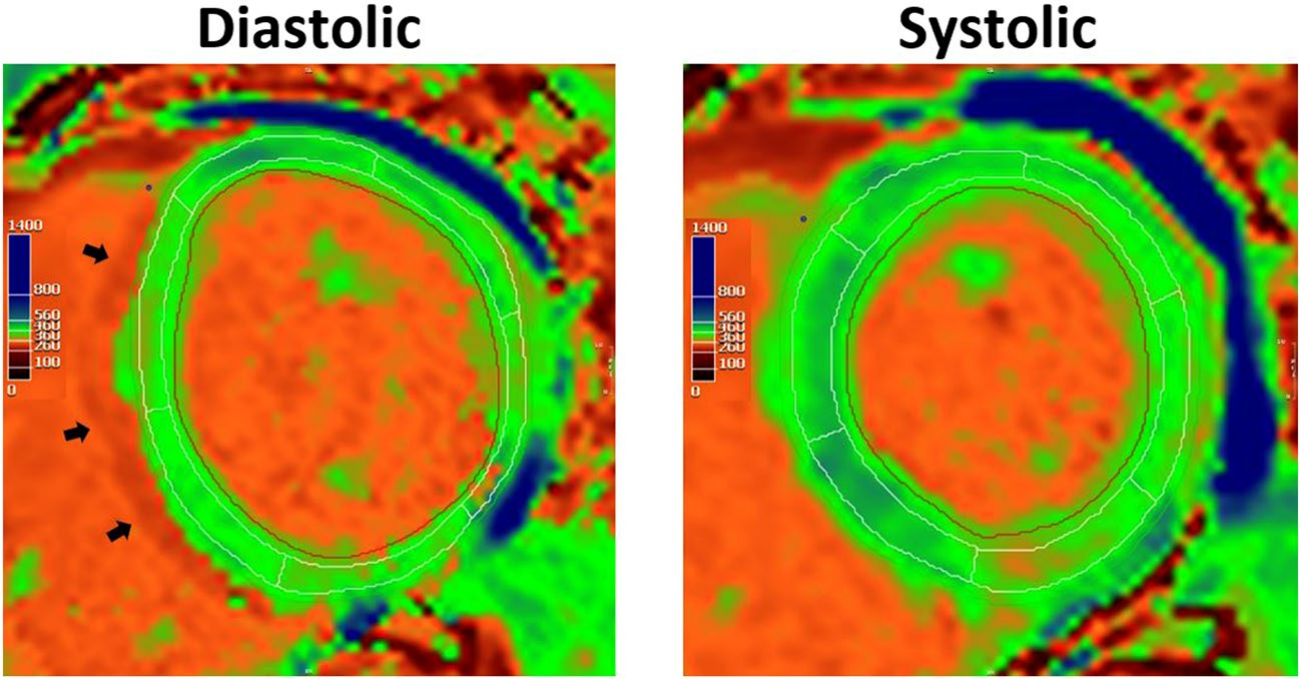
Diastolic and systolic T1 maps, analysed with 20% offset of endocardial and epicardial contours, split into six American Heart Association segments. Suspicions for diastolic mapping gating artefact is raised by the presence of a subtle “double” septal border (black arrows), which can be visualised when comparing the individual acquisition images (Video 1). R^2^ in systole was higher in 62% of cases, whilst diastolic measurement was higher in 32% (6% identical).

Contrary to our hypothesis, this study found systolic imaging generated similar T1 and ECV values when compared to diastolic imaging. It is worth noting that compared to systolic acquisition, diastolic T1 mapping was more prone to artefact (example shown in Fig. 4), resulting in a higher segment exclusion rate. Pertinently, if the screening and exclusion of artefactual segments were not performed as meticulously within daily clinical practice, practitioners may derive clinically significant errors in T1 and ECV values, which appear to be less frequent with systolic imaging.

**Figure 5:**
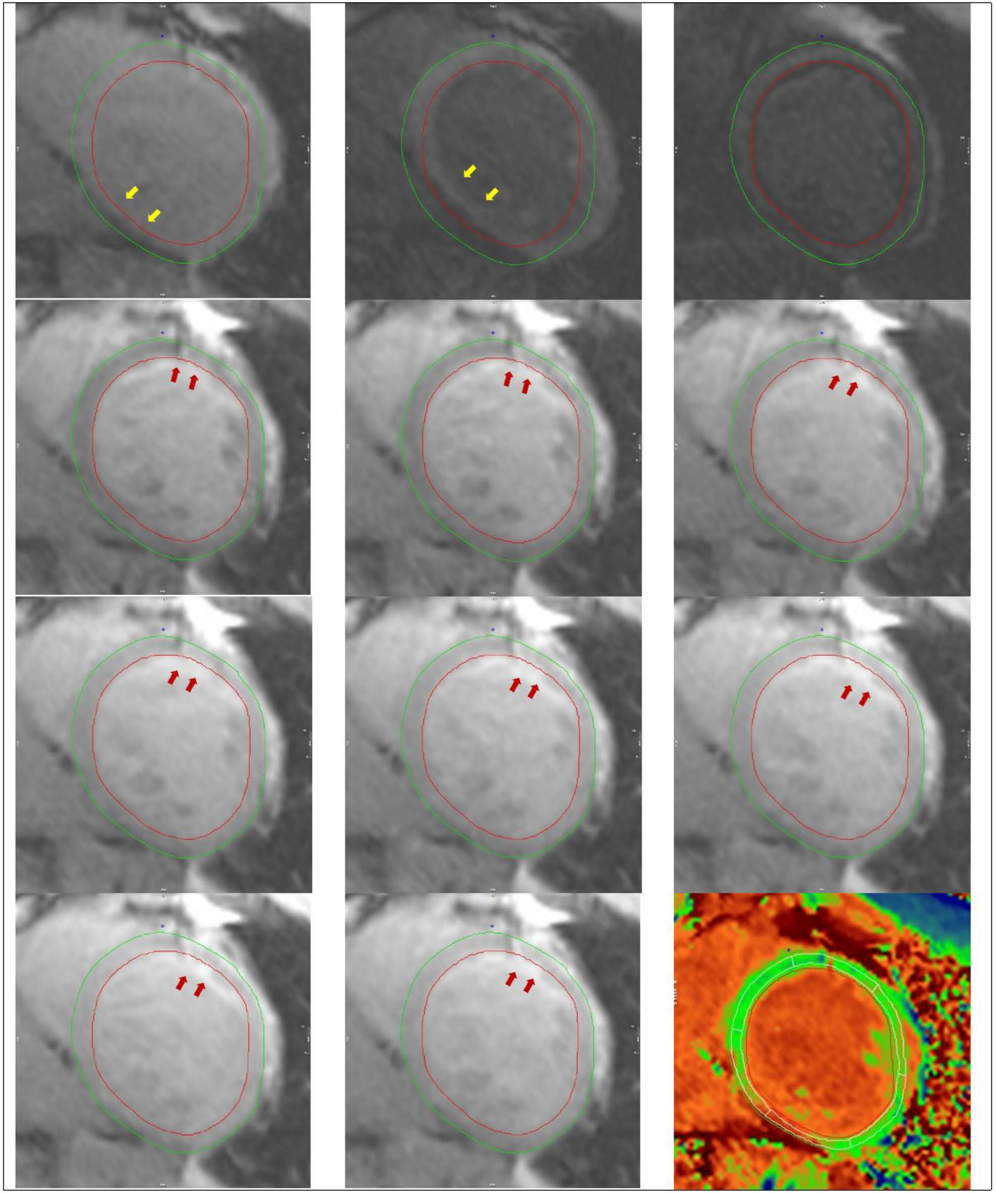
Illustration of (1) susceptibility artefact originating from the epicardial fat (red arrows), and (2) motion correction artefact (yellow arrows) on individual MOLLI acquisition images, with their resulting effects on the T1 map (bottom right). The position and shape of epicardial and endocardial contours across acquisiton images have been kept identical to help highlight myocardial motion.

Even following the exclusion of a higher number of artefact-affected segments during diastolic T1 mapping, systolic imaging was less variable (higher R^2^ values) in all patients and subgroups (males, females, sinus rhythm and AF patients). In this respect, systolic and diastolic R^2^ values showed strong correlation (r = 0.81) with less variability (SD = 0.8) between the methods than in the measurements themselves (SD = 1.4), explaining the statistical significance despite small mean differences. Our rationale for better R^2^ values with systolic imaging is that this is likely related to reduction in susceptibility to gating artefact. A typical example in a patient with atrial fibrillation is illustrated in Supplementary Video S1, with the corresponding T1 maps presented in Fig. 5. However, more subtle differences in R-R interval may occur even in patients with sinus rhythm, causing a “blurring” of the myocardial-blood pool interface that is incompletely compensated using a standard 20% endocardial- and epicardial-offset.

Extracellular volume expansion has been reported in patients with atrial fibrillation; a phenomenon which we hypothesized might be in part due to blood pool contamination when performing diastolic T1 mapping in atrial fibrillation patients. Similarly, female gender has been previously linked with higher ECV and native T1 values, possibly due to higher myocardial blood pool volume^8,9^. We therefore hypothesised that systolic T1 mapping would help to equalise the male–female divide as cardiac contraction should reduce the myocardial blood volume as blood is squeezed out of the cardiac vasculature^16^. However, our data failed to support our hypothesis, with diastolic and systolic T1 mapping producing similar ECV and T1 values.

Diastolic variation in T1 and ECV values have previously been reported between sexes^7,9^ and those with AF in diastole^17^. This study found that the same trends are observed in systole, whereby females and patients in AF have higher ECV fractions and native T1 values compared to males and patients in sinus rhythm respectively. Although absolute differences are small, the significantly lower variability with systolic T1 mapping supports use in the clinical setting.

While previous studies have highlighted differences in T1 time and ECV during the cardiac cycle, these studies have been limited to small sample sizes of mostly healthy volunteers^6,18,19^. To date, this study is the first to evaluate the robustness of systolic T1 mapping in a larger sample size of patients with mitral regurgitation and permanent AF. The differences observed in this study are likely due to the lower sensitivity to R-R variability, arrhythmias, and partial-volume effects with systolic readout when myocardial muscle to blood volume ratio is greatest. This effect was expected to be greatest in females due to their thinner myocardium, as supported by data from Ferreira et al*.*^19^, however the present study found significant higher R^2^ coefficient values in males also.

## Limitations

The authors acknowledge the limitations of this single centre study. In particular, subgroup analysis for females and patients in AF involved relatively small sample populations, and so basal and mid-ventricular values of each patient were analysed as separate data-points. Additionally, reproducibility of T1 mapping measures were assessed in systole and diastole but repeated measures of T1 mapping on separate scans (patient on and off table) were not performed.

Different T1 mapping sequences have different susceptibilities to artefact/error, so these findings may not be applicable to all T1 mapping sequences. However, errors due to blood pool contamination are common to all sequences and it is likely that our findings are relevant to most or all of these sequences. Furthermore, the analysis may be impacted by low image signal-to-noise ratio, artefacts, varying slice locations and small dataset size.

## Conclusion

Myocardial T1 mapping is associated with similar T1 and ECV values in systole and diastole. Systolic acquisition is associated with reduced susceptibility to artefact and lower variability due to greater myocardium:blood volume ratio and less gating artefact.

## Supplementary Information

Supplementary Information 1.

Supplementary Video 1.

## Data Availability

The datasets used and/or analysed supporting the conclusions of the article are available from the corresponding author on reasonable request.
