# Supplementary material for: Left ventricular T1-mapping in diastole versus systole in patients with mitral regurgitation
Source: Sci Rep. 2022 Nov 21;12:20000. doi: 10.1038/s41598-022-23314-6 (PMC9678898; doi:10.1038/s41598-022-23314-6)
Supplement: Supplementary file 1 — Supplementary Information 1. [file 41598_2022_23314_MOESM1_ESM.docx]

|  |  |  | Mean + SD |  | Lower | Upper |  |
| --- | --- | --- | --- | --- | --- | --- | --- |
| All | Native T1 (ms) | 103 | 978 ± 34 | 975 ± 28 | -1.56 | 7.64 | 0.193 |
|  | Post-contrast T1 (ms) | 100 | 468 ± 36 | 471 ± 33 | -7.42 | 0.63 | 0.097 |
|  | ECV (%) | 98 | 25.9 ± 3.4 | 25.8 ± 2.5 | -0.01 | 0.01 | 0.737 |
|  | R^2^ | 206 | 96.1 ± 1.4 | 96.3 ± 1.4 | -0.36 | -0.13 | <0.001 * |
| SR | ECV fraction (%) | 79 | 25.6 ± 3.6 | 25.5 ± 2.5 | -0.01 | 0.01 | 0.788 |
|  | Native T1 (ms) | 83 | 974 ± 33 | 974 ± 28 | -4.63 | 4.83 | 0.966 |
|  | Post-contrast (ms) | 81 | 469 ± 35 | 472 ± 31 | -6.42 | 1.32 | 0.194 |
|  | R^2^ | 164 | 96.1 ± 1.4 | 96.4 ± 1.4 | -0.33 | -0.07 | 0.003 * |
| AF | ECV fraction (%) | 19 | 27.2 ± 2.6 | 27.1 ± 2.0 | -0.01 | 0.01 | 0.829 |
|  | Native T1 (ms) | 20 | 996 ± 35 | 980 ± 27 | 2.36 | 28.08 | 0.023 |
|  | Post-contrast (ms) | 19 | 461 ± 41 | 468 ± 44 | -21.32 | 7.30 | 0.317 |
|  | R^2^ | 40 | 95.6 ± 1.3 | 96.0 ± 1.2 | -0.64 | -0.11 | 0.007 * |

**Supplementary Table 1: Mid-septal region-of-interest T1 mapping in AF and sinus rhythm: differences between diastole and systole. P values with a * denotes statistical significance**

| **Mean absolute bias**  **(n=20 T1 maps)** | **Diastole (ms)** | **Systole (ms)** | **P value** |
| --- | --- | --- | --- |
| Intra-observer | 10.9 + 8.4 | 7.1 + 5.9 | 0.076 |
| Inter-observer | 9.4 + 10.3 | 6.4 + 4.1 | 0.121 |

**Supplementary Table 2: Demonstrating mean absolute bias in native T1 times to assess intra-observer and inter-observer variability**

**
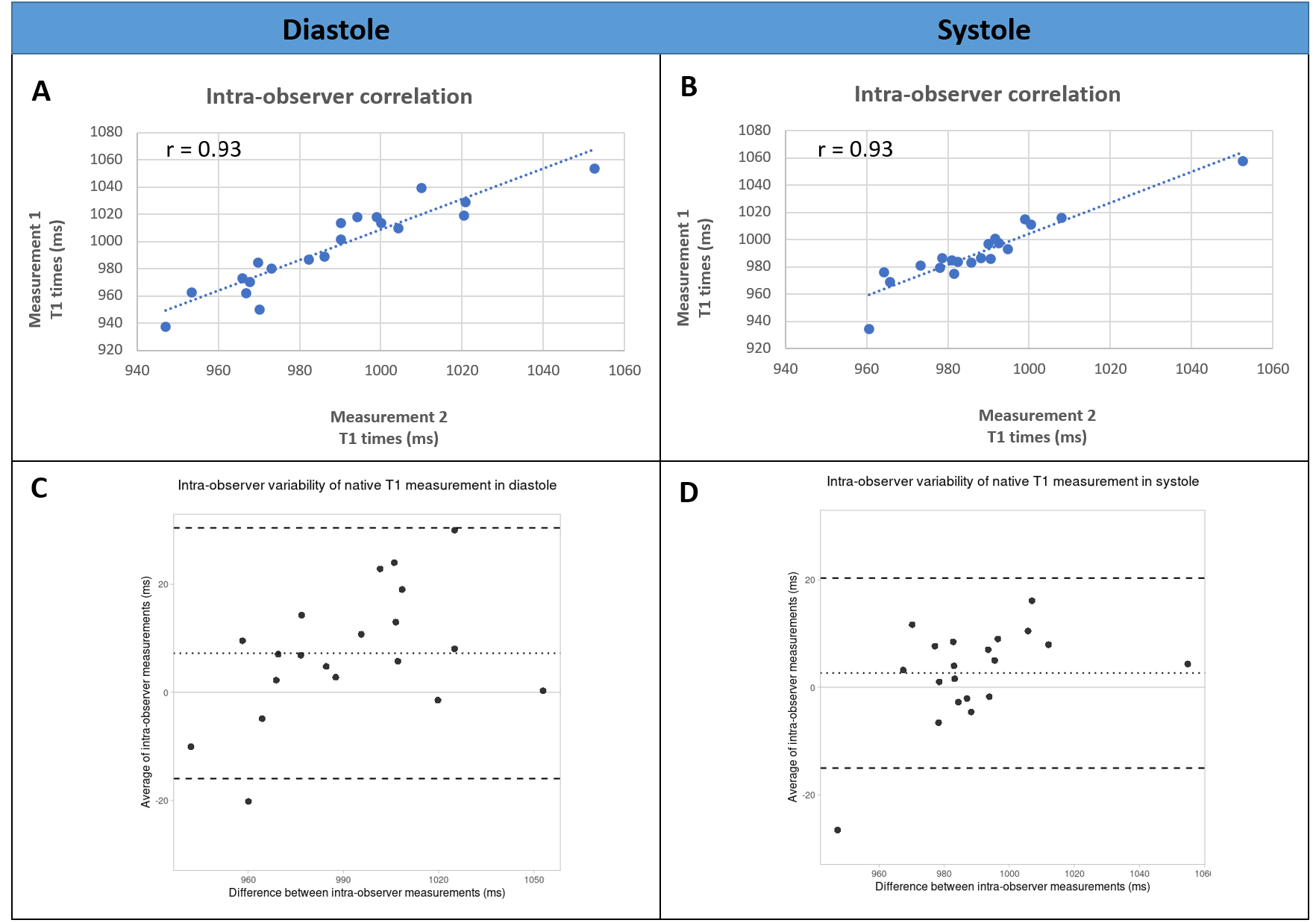
**

**Supplementary Figure 1: Demonstrating intra-observer variability of native T1 times in diastole (1A and 1C) and systole (1B and 1D). Figures 1A and 1B demonstrate good correlation between measurements 1 and 2 in both diastole and systole (Pearson’s correlation coefficient 0.93). Figures 1C and 1D represent Bland-Altman plots. The dotted line indicates the average difference and the dashed lines indicate the limits of agreement.**

**
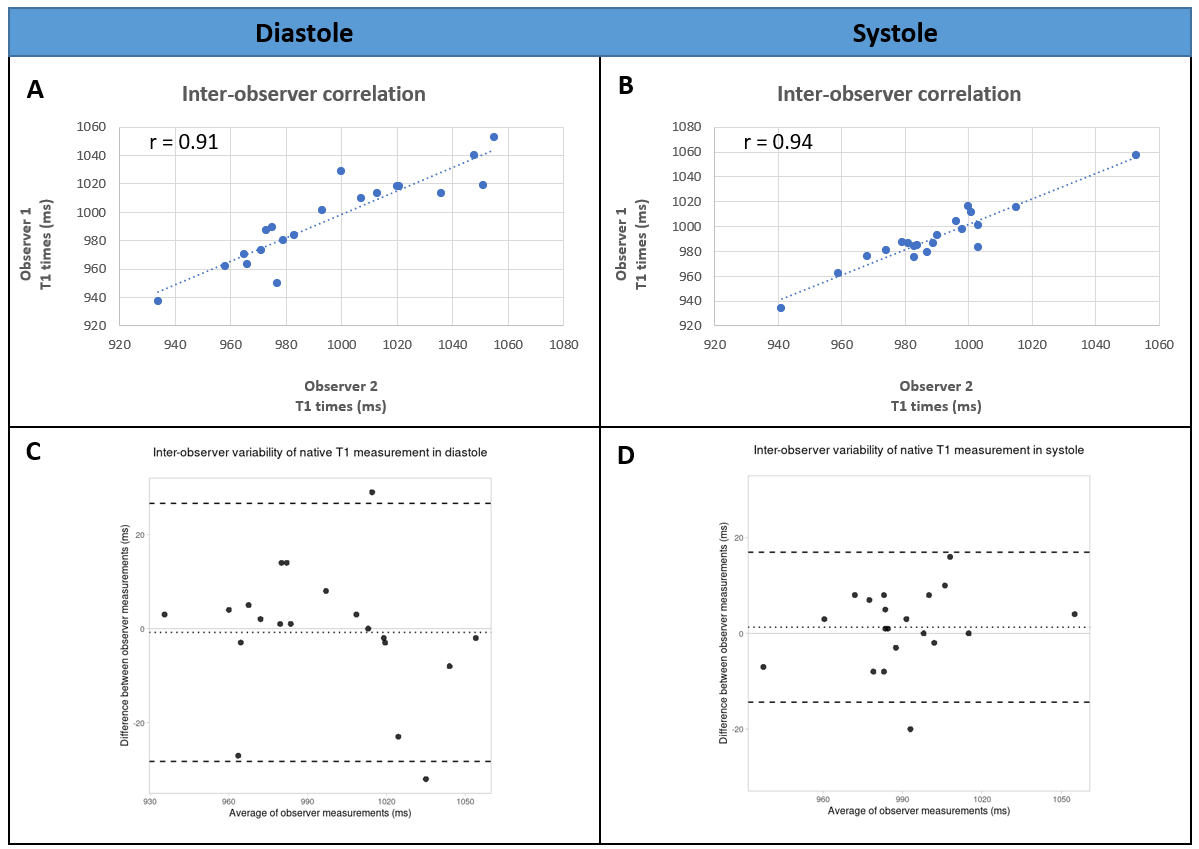
**

**Supplementary Figure 2: Demonstrating inter-observer variability of native T1 times in diastole (2A and 2C) and systole (2B and 2D). Figures 2A and 2B demonstrate good correlation between observers in both diastole and systole (Pearson’s correlation coefficient 0.91 and 0.94 respectively). Figures 2C and 2D represent Bland-Altman plots. The dotted line indicates the average difference and the dashed lines indicate the limits of agreement.**

**Supplementary Video S1 legend:** An example of a patient with atrial fibrillation with susceptibility to gating artefact in diastole (right) compared to systole (left)
